# P-1043. Interdisciplinary Initiative for Sustained CAUTI Reduction

**DOI:** 10.1093/ofid/ofaf695.1238

**Published:** 2026-01-11

**Authors:** Margo Leavitt, Alex Woods, Jessica R Miller, Bianca Johns, Julie Gregory, Sandra Williams, James Newton

**Affiliations:** Washington Regional Medical Center, Fayetteville, AR; Washington Regional Medical Center, Fayetteville, AR; Washington Regional Medical Center, Fayetteville, AR; Washington Regional Medical Center, Fayetteville, AR; Washington Regional Medical Center, Fayetteville, AR; Washington Regional Medical Center, Fayetteville, AR; Washington Regional Medical Center, Fayetteville, AR

## Abstract

**Background:**

Catheter-associated urinary tract infections (CAUTIs), one of the most common nosocomial infections, are correlated with increased mortality, prolonged hospital stays, and excess healthcare costs. CAUTI incidence in our 425-bed acute care hospital exceeded 1.2 per 1,000 device days. Existing prevention strategies, including root cause analysis and a maintenance bundle had previously failed to impact CAUTI rates.

**Figure:**
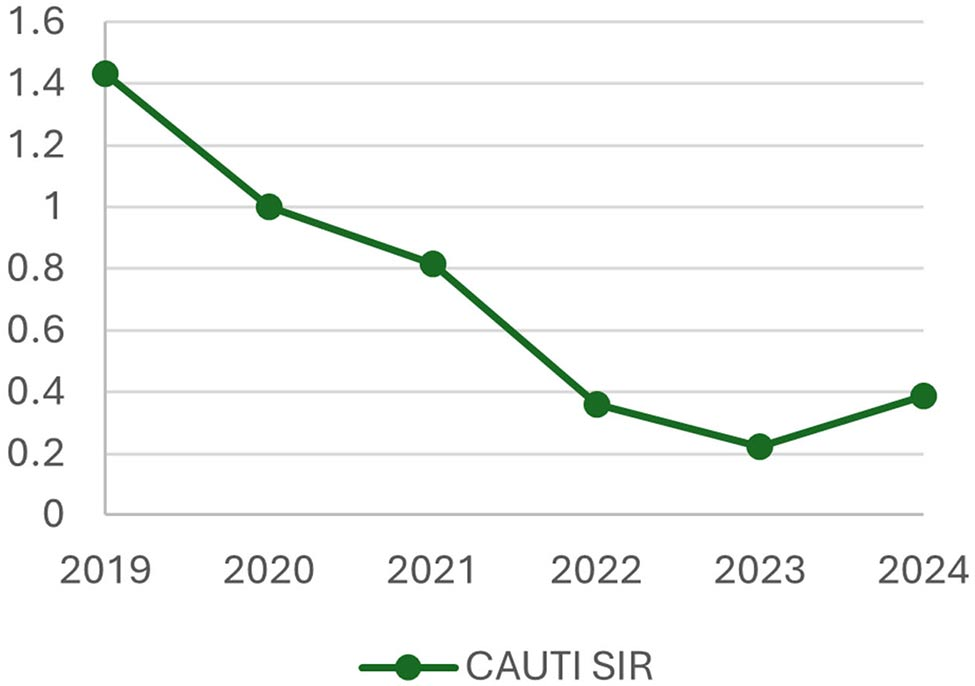

**Figure:**
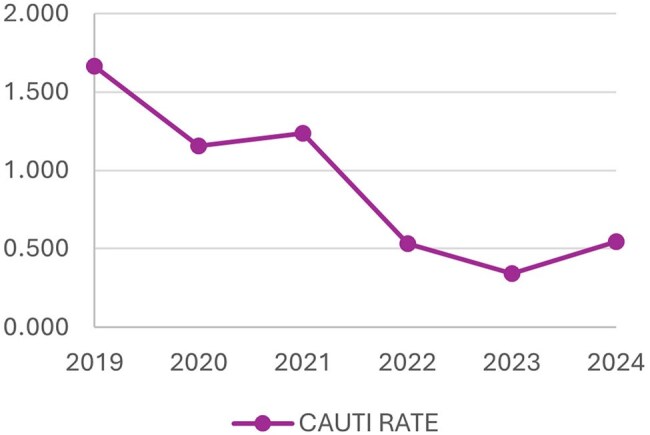

**Methods:**

Our facility initiated a collaborative CAUTI prevention quality improvement project, with dedicated ID physician and nursing champions. Areas for improvement included decreasing urinary catheter utilization, optimizing appropriate urine sampling processes, and inconsistent assessment of urinary catheter necessity. Daily audits of foley catheter indication and overall utilization rates are conducted by the champions, with real-time feedback given to providers, leadership, and nursing staff. The team developed algorithms for nurse-driven retention voiding trials and evidence-based external catheter indications to allow for earlier removal of indwelling catheters. To prevent false positive urine culture results, we developed guidelines and administrative controls to ensure urine cultures were obtained from catheters that have been in place for less than 48 hours.

**Figure:**
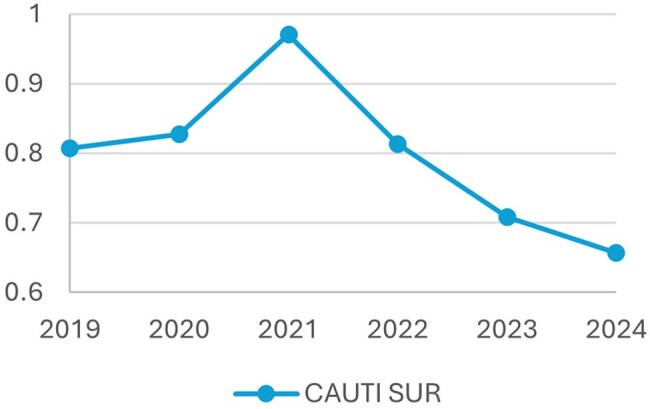

**Results:**

After implementation, the CAUTI rate decreased from 1.2 to 0.53 per 1,000 device days (a 57% reduction), with sustained reduction in the subsequent two years. Similarly, the standardized infection ratio (SIR), as calculated by the National Healthcare Safety Network, decreased from 0.816 to 0.387, nadir of 0.224 in 2023. Furthermore, indwelling catheter utilization decreased, as demonstrated by a 32% standardized utilization ratio (SUR) reduction.

**Conclusion:**

By emphasizing interdisciplinary cooperation, real-time feedback, and administrative controls to support best practices, our facility achieved and has sustained a substantial reduction in CAUTI metrics. Intervention practices remain in place and our facility continues to examine avenues to integrate these practices into our culture.

**Disclosures:**

All Authors: No reported disclosures

